# Subnormal short‐latency facial mimicry responses to dynamic emotional facial expressions in male adolescents with disruptive behavior disorders and callous‐unemotional traits

**DOI:** 10.1111/psyp.13945

**Published:** 2021-09-22

**Authors:** Anton van Boxtel, Ruud Zaalberg, Minet de Wied

**Affiliations:** ^1^ Department of Cognitive Neuropsychology Tilburg University Tilburg The Netherlands; ^2^ Wageningen University & Research, Biometris Wageningen The Netherlands; ^3^ Department of Youth and Family Faculty of Social and Behavioral Sciences Utrecht University Utrecht The Netherlands

**Keywords:** callous‐unemotional traits, disruptive behavior disorders, EMG, emotional facial expressions, facial mimicry

## Abstract

Using still pictures of emotional facial expressions as experimental stimuli, reduced amygdala responses or impaired recognition of basic emotions were repeatedly found in people with psychopathic traits. The amygdala also plays an important role in short‐latency facial mimicry responses. Since dynamic emotional facial expressions may have higher ecological validity than still pictures, we compared short‐latency facial mimicry responses to dynamic and static emotional expressions between adolescents with psychopathic traits and normal controls. Facial EMG responses to videos or still pictures of emotional expressions (happiness, anger, sadness, fear) were measured. Responses to 500‐ms dynamic expressions in videos, as well as the subsequent 1500‐ms phase of maximal (i.e., static) expression, were compared between male adolescents with disruptive behavior disorders and high (*n* = 14) or low (*n* = 17) callous‐unemotional (CU) traits, and normal control subjects (*n* = 32). Responses to still pictures were also compared between groups. EMG responses to dynamic expressions were generally significantly smaller in the high‐CU group than in the other two groups, which generally did not differ. These group differences gradually emerged during the 500‐ms stimulus presentation period but in general they were already seen a few hundred milliseconds after stimulus onset. Group differences were absent during the 1500‐ms phase of maximal expression and during exposure to still pictures. Subnormal short‐latency mimicry responses to dynamic emotional facial expressions in the high‐CU group might have negative consequences for understanding emotional facial expressions of others during daily life when human facial interactions are primarily dynamic.

## Abstract 2

During human interactions, short‐latency facial mimicry responses occur to dynamic emotional facial expressions of others. These are preconscious, automatic responses which cannot be voluntarily controlled. They may be important for emotional understanding of others and appear to be subnormal in male adolescents with psychopathic traits.

## 1 INTRODUCTION

Children and adolescents with disruptive behavior disorders (DBD), including the DSM‐5 categories of oppositional defiant disorder (ODD) and conduct disorder (CD), exhibit various forms of antisocial behavior. The behaviors of ODD (e.g., being angry, blaming others, arguing with adults) are typically of a less severe nature than the behaviors of CD (e.g., physical cruelty, lying, vandalism), but both are associated with significant impairments in social, academic, or occupational functioning (American Psychiatric Association, 2013). Developmental pathways leading to DBD are varied, even within each diagnostic category (Viding & McCrory, 2020). Callous‐unemotional (CU) traits designate a particularly problematic subgroup of DBD individuals with distinct emotional, cognitive, and biological characteristics (Blair, 2013; Blair et al., 2014; Frick & Kemp, 2021; Frick et al., 2014; Salekin, 2017), exhibiting more severe and stable patterns of delinquency (Reidy et al., 2017), and running the risk to develop psychopathy in adulthood (McMahon et al., 2010). CU traits, including lack of empathy, lack of guilt, and low emotional responsiveness, represent the affective dimension of child and adult psychopathy (Frick & Hare, 2001). A recent meta‐analysis of the relationships between CU traits and questionnaire measures of trait‐like empathy demonstrated moderate negative relationships between CU traits and measures of both affective and cognitive empathy (Waller et al., 2020). Yet, studies examining aspects of state‐like empathy have shown more consistent deficiencies in affective than in cognitive empathy in DBD individuals with CU traits (Blair, 2013; Frick & Kemp, 2021; Pijper et al., 2017; Viding & McCrory, 2019). Focusing more closely on emotional responsiveness in youth with conduct problems and CU traits, Northam and Dadds (2020) demonstrated that individuals with high compared to low levels of CU traits are less emotionally responsive, though results were fairly inconsistent. More consistent differences between subtypes were found in studies using older samples (adolescents vs. children), studies using physiological measures (rather than behavioral or self‐report measures), and studies using stimuli evoking other‐oriented emotions (i.e., affective empathy) rather than self‐oriented emotions.

Affective empathy is supposed to be accompanied by primitive or motor empathy in the form of automatic mimicry responses synchronizing emotional facial expressions, vocalizations, postures, and movements of others (Blair, 2005). According to Blair, no definite conclusions can be drawn regarding the role of motor empathy in persons with psychopathic traits since no studies have formally assessed motor empathy in psychopathy. In a preliminary study in a nonclinical student population using automated computerized coding of facial emotional expressions, individuals high on psychopathic traits showed less motor empathy when exposed to negative facial emotional expressions of others than individuals low on psychopathic traits (Khvatskaya & Lenzenweger, 2016). Adolphs (2006) hypothesizes that rapid automatic mimicry responses contribute to recognition of others' emotional facial expressions and may be crucial for the emergence of affective empathy.

Facial mimicry processes may be considered a determinant of emotional contagion (also called empathic arousal; Waller et al., 2020), that is “the cognitive ability to intuit what another person is feeling” (Hatfield et al., 1994, 2009). However, the role of facial mimicry as an essential link in the transmission of basic emotional states from one person to another, and thus its role in emotional contagion and subsequent emotion recognition, is not unequivocal. As apparent from various studies (e.g., Blairy et al., 1999; Hess & Blairy, 2001; Lischetzke et al., 2020; Olszanowski et al., 2020), facial mimicry may be involved in emotional contagion but its role may vary between individuals and may also depend on situational factors such as whether observed emotional expressions are strong or weak, are realistic or posed, can be easily recognized or not, represent a stereotypical emotion or a more complex emotional state, a positive or negative emotion, or a repulsed or non‐repulsed emotional response. In addition, in experimental studies facial mimicry may be dependent on whether still pictures of emotional facial expressions or dynamic expressions with higher ecological validity are used as stimuli.

Whether facial mimicry occurs in daily life will primarily be determined by the social context, implying that mimicry does not automatically occur (Fischer & Hess, 2017; Hess, 2020). As elucidated by these authors, emotional expressions will be mimicked if expressor and mimicker share the perspective that gave rise to the emotion. The emotional or social meaning of facial expressions may be mimicked rather than facial features as such. Mimicry is thus not a priori an automatic process but may be considered a controlled process determined by social interactions. It will not only be determined by positive but also by negative affiliative connections between persons. Affiliative emotions, such as happiness or sadness, may be mimicked stronger than non‐affiliative expressions like anger or disgust. Mimicry is thus primarily dependent on the goal to affiliate with others and not on observing facial motor activities of others. Also factors like competition, conflict, ingroup membership, social power, and personality traits may determine if mimicry strictly occurs. This would imply that in many social contexts mimicry cannot be considered a necessary determinant of emotion recognition.

Nevertheless, besides such social mimicry responses with relatively long response latencies, short‐latency automatic mimicry responses occur which might have diagnostic value for psychopathy. In healthy persons, passively viewing static or dynamic emotional facial expressions induces short‐latency facial mimicry responses. EMG responses with latencies shorter than 500 ms were observed in corrugator supercilii and zygomaticus major muscles following presentations of angry or happy expressions, respectively (Achaibou et al., 2008; de Wied et al., 2006; Dimberg, 1997; Dimberg & Thunberg, 1998). Such short‐latency responses may be considered automatic responses since they are difficult to control voluntarily by advertent suppression or contracting facial muscles with an incongruent action (Dimberg et al., 2002). Such automatic responses also occurred when emotional expressions were not recognized because stimuli were presented only a few tens of milliseconds and backward masked by a neutral stimulus (Bailey & Henry, 2009; Dimberg et al., 2000). This suggests that short‐latency facial mimicry responses are automatic, preconscious responses.

The amygdala might play an important role in short‐latency facial mimicry responses. It is involved in the earliest, automatic responses to emotional facial expressions, in particular dynamic expressions (Adolphs, 2002). Such stimuli may reach the amygdala across different fast‐conducting pathways, being either a direct subcortical pathway from the retina across superior colliculus and pulvinar, or fast‐conducting pathways across early striate or extrastriate visual cortical areas (Garvert et al., 2014; Pessoa & Adolphs, 2010; Tamietto & De Gelder, 2010). Anyway, the amygdala is assumed to play an important role in various short‐latency networks involving subcortical and cortical visual areas (Johnson, 2005; Pessoa & Adolphs, 2010). Within these trajectories, response latencies of the amygdala to emotional facial expressions are shorter than 100 ms (Diano et al., 2017; Méndez‐Bértholo et al., 2016) because it is activated by fast‐conducting magnocellular cells, either through the trajectory across superior colliculus and pulvinar or through fast cortical channels (Tamietto & De Gelder, 2010; Vuilleumier, 2005). Therefore, the amygdala quickly responds to low spatial frequency information in facial expressions and thus responds to more coarse aspects of the expression like actions around eyes and mouth which are prominent in emotional expressions (Adolphs, 2008; Johnson, 2005; Vuilleumier et al., 2003; Whalen et al., 2004).

Rapid activation of this fast amygdalar route majorly has preconscious effects. In healthy persons or patients with cortical blindness it did not lead to conscious experience of emotional stimuli (Tamietto & De Gelder, 2010). This route was also activated when emotional faces were backward masked (Adolphs, 2008; Killgore & Yurgelun‐Todd, 2004; Whalen et al., 1998). Patients with cortical blindness or contralateral neglect were able to recognize emotional facial expressions when stimuli were presented to the blind half of the visual field (Adolphs, 2003a; Pegna et al., 2005). They showed similar mimicry responses to these stimuli and stimuli presented to the intact half of the field (Tamietto et al., 2009). Low spatial frequency information also underlies most visual abilities in infants who can detect coarse facial and emotional cues in the absence of a mature cortical visual system (Vuilleumier et al., 2003).

The amygdala is particularly involved in spontaneous emotional responses of muscles in the upper part of the face like frontalis, corrugator supercilii, and orbicularis oculi. Contrary to lower facial muscles, upper facial muscles are less susceptible to voluntary control by neurons in the contralateral primary motor or premotor cortex and are less well represented in these cortical areas than lower facial muscles (Rinn, 1984). They are largely controlled by direct ipsilateral and contralateral projections from a motor region within the anterior cingulate cortex (Morecraft et al., 2001). This region receives projections from the lateral and accessory basal nuclei of the amygdala (Morecraft et al., 2007). It specifically subserves spontaneous, involuntary emotional expressions and functions independently of voluntary actions being controlled by the primary motor or premotor cortex (Cattaneo & Pavesi, 2014). The voluntary cortical motor system cannot affect a genuine spontaneous emotional motor response. This may explain why spontaneous mimicry responses to emotional facial expressions are difficult to control voluntarily (Dimberg et al., 2002). The amygdalar route across anterior cingulate obviously provides short‐latency responses in facial muscles. In patients with occipital lobe lesions, the photic blink reflex recorded in orbicularis oculi showed an EMG response latency of 50 ms irrespective of whether the light flash was presented to the blind or sighted visual hemifield (Hackley & Johnson, 1996), suggesting the involvement of the fast processing route across the amygdala.

Although there exists a long‐standing discussion whether the amygdala primarily responds to negative facial emotions, particularly fear, more recent studies in healthy persons show that during passive exposure it responds to all negative and positive basic emotions (Adolphs, 2010; Costafreda et al., 2008; Fusar‐Poli et al., 2009; Sergerie et al., 2008). Nevertheless, fearful expressions generally elicited larger responses in central or basolateral amygdalar nuclei than angry or happy faces (Costafreda et al., 2008; Whalen et al., 2001). Compared with healthy persons, patients with amygdala damage were generally impaired in recognition of negative emotions, particularly, but not exclusively, fear (Adolphs, 2003b; Rapcsak et al., 2000; Zald, 2003). This deficit may be related to problems with fast, automatic processing of the eye region without sensory awareness which may be important for rapid detection of threatening situations being characterized by expressions of fear or anger (Adolphs, 2008; Diano et al., 2017; Rotshtein et al., 2010).

In persons with psychopathic traits, impaired recognition of emotional facial expressions occurs. Particularly expressions of fear, sadness, or anger were worser recognized by (generally male) adults (Blair et al., 2004), adolescents (Fairchild et al., 2009; Muñoz, 2008), or children (Blair & Coles, 2000; Blair et al., 2001; Stevens et al., 2001) with such traits. Fear‐recognition deficits in children with these traits could also be demonstrated using a paradigm testing automatic, preconscious detection of emotional facial expressions (Sylvers et al., 2011), suggesting that deficits are not caused by a lack of conscious attention to emotionally salient cues. Although many studies focused on the relationship between psychopathy and poor recognition of fear or sadness, a meta‐analysis of studies in adults, adolescents, or children with psychopathy also showed deficits in recognition of happiness, surprise, anger, and disgust (Dawel et al., 2012). In all studies mentioned here, including those in the meta‐analysis, presentation times of expressions were relatively long (at least 1 s) so that a specific role of early processing stages in impaired recognition could not be evaluated.

The question is whether the amygdala is involved in such recognition deficits. Problems with recognition of distress signals like fearful or sad expressions in youths and adults with psychopathic traits may be related to amygdala dysfunction already existing at a young age (Blair, 2013). Reduced amygdala responses to negative facial expressions were observed in both adults, adolescents, and children with psychopathic tendencies (Blair, 2010; Decety et al., 2014; Jones et al., 2009; Marsh et al., 2008; Passamonti et al., 2010). Although also in these studies presentation times of expressions were relatively long (at least 1 s), in a few studies subnormal amygdala responses obviously occurred at a pre‐attentive level. In children with high CU traits, backward‐masked unrecognized facial expressions of fear being presented for 17 ms induced an abnormally low amygdala response (Viding et al., 2012). Another study performed in youths under conditions of low attentional load found that higher CU traits were related to smaller amygdala responses to fearful expressions being presented for 200 ms (White et al., 2012). In these two studies, high CU traits were thus obviously related to subnormal early amygdala responses to fearful expressions.

As indicated above, the fast route between amygdala and motoneurons of upper facial muscles (cf. Morecraft et al., 2001, 2007) would enable short‐latency facial mimicry responses to emotional facial expressions. Several studies suggest that this route may be differently involved in dynamic and static emotional expressions. In healthy persons, effects of emotional facial expressions on facial mimicry responses, amygdala responses, and emotion recognition differed between dynamic expressions, morphed pictures, and still pictures. Mimicry responses to dynamic expressions of anger, fear, disgust, or happiness were larger than responses to still pictures (Krumhuber et al., 2013; Rymarczyk et al., 2011, 2016; Sato et al., 2008) and showed stronger relationships with experienced negative or positive emotional valence (Sato et al., 2013). Dynamic expressions of fear (Sato et al., 2004), anger, or happiness (Arsalidou et al., 2011) also elicited larger amygdala responses than static expressions. Dynamic expressions like those presented in video clips have several advantages compared with morphed pictures which show linear changes across time within all locations of the face. The natural, nonlinear unfolding of dynamic expressions has a higher ecological validity than morphed pictures and resulted in faster and more accurate emotion recognition as well as higher judgements of experienced emotion intensity (Calvo et al., 2016; Krumhuber et al., 2013).

In the current study we investigated whether adolescents with DBD and high or low CU traits showed abnormalities in short‐latency mimicry responses, particularly to dynamic emotional facial expressions. Dynamic expressions may have large ecological validity since during normal human interactions facial expressions are predominantly dynamic. We used 500‐ms video clips, during which expressions increased from neutral to maximal intensity. Earlier work using morphed pictures showed that the rate of change of pictures influenced rated intensity and naturalness of perceived expressions (Kamachi et al., 2001; Sato & Yoshikawa, 2004; Yoshikawa & Sato, 2008). For expressions of basic emotions, experienced intensity and naturalness became generally higher with a higher rate of change. The optimal duration of expressions was in the range of 500–740 ms for fear, happiness, and anger but appeared to be somewhat longer (900–1000 ms) for sadness (Hoffmann et al., 2010; Sato & Yoshikawa, 2004). Kinematic manipulation of facial expressions of happy, angry, and sad emotional states confirmed that emotion recognition becomes better with increasing speed of angry or happy expressions and with decreasing speed of sad expressions (Sowden et al., 2021). The major question of our study was whether adolescents with high CU traits showed abnormal short‐latency mimicry responses to dynamic emotional expressions. For this purpose we analyzed facial EMG responses during subsequent 100‐ms intervals following onset of 500‐ms video clips.

## 2 METHOD

The Medical Ethical Committee of the University Medical Center Utrecht approved the study protocol and both parents and adolescents gave written consent prior to participation.

### 2.1 Participants

The current data were collected during an experimental session in which also responses to empathy‐inducing film clips were investigated which were earlier reported (de Wied et al., 2012). Participants, their recruitment, diagnostic and psychometric characteristics, and inclusion criteria are extensively described in this earlier report. A group of 31 male adolescents (aged 12–15 years) with DBD as set out in the DSM‐IV‐TR (American Psychiatric Association, 2000), including 17 participants with ODD and 14 with CD, participated in this study. They were recruited from special schools for adolescents with severe behavioral problems. The presence of ODD or CD was assessed using the parent version of the Diagnostic Interview Schedule for Children (DISC‐IV, Dutch version) (Ferdinand & van der Ende, 2000). Thirty‐two Male normal control (NC) adolescents were recruited from a regular school.

Parents and teachers of all participants completed the Child Behavior Checklist (CBCL/4‐18; Achenbach, 1991a) and Teacher's Report Form (TRF/4‐18; Achenbach, 1991b), respectively. DBD adolescents obtained significantly higher scores than controls on the CBCL and TRF externalizing and internalizing scales, which confirmed the presence of group differences in conduct problems (see de Wied et al., 2012 for details).

Parents and teachers also completed the Antisocial Process Screening Device (APSD) (Frick & Hare, 2001), in the Dutch translation (de Wied et al., 2014). The APSD is a 20‐item questionnaire designed to measure psychopathic traits in children and adolescents. This scale includes three factors: Callous‐Unemotional (six items), Narcissism (seven items), and Impulsivity (five items). The intercorrelations between parents and teachers for the three dimensions were: *r*_CU_ = .46, *p* < .01; *r*_Narcissism_ = .52, *p* < .01; *r*_Impulsivity_ = .51, *p* < .01. Ratings from parents and teachers were combined by using the highest score for each item (cf. Frick & Hare, 2001). The CU dimension, capturing the callous interpersonal style that is critical to the construct of psychopathy, was used to assign DBD adolescents to groups with high (CU+; *n* = 14) or low (CU−; *n* = 17) CU traits. Because Narcissism and Impulsivity were highly correlated in our study (*r* = .83, *p* < .001), they were combined to form the I/CP factor, reflecting impulsivity and conduct problems. Internal consistency was acceptable for the CU dimension (*α* = .71) and good for the I/CP dimension (*α* = .93).

As we earlier reported (de Wied et al., 2012), the CU+ group obtained significantly higher scores than the CU− group or control group on the APSD total score and CU and I/CP dimensions. The CU− group, in turn, obtained higher scores than controls on the APSD total score and I/CP dimension but not on the CU dimension. There were no group differences in age or intelligence.

### 2.2 Emotional facial stimuli

Videos of dynamic facial expressions of four different emotions (happiness, anger, sadness, fear) were presented. Two female and two male actors (aged 15–25 years) were trained to produce these expressions according to a fixed time schedule. Videos had a duration of 5.5 s and consisted of five contiguous stages: (1) a 2‐s still picture of the actor's neutral face; (2) a 500‐ms dynamic expression, increasing from neutral to maximal expression; (3) maintaining maximal expression for 1500 ms; (4) a 500‐ms relaxation stage; (5) a 1‐s still picture of the neutral face. During the training, the actors were assisted by a moving time bar at the bottom of the computer screen indicating the duration of the subsequent stages in different colors. The emotional expressions were based on the Facial Action Coding System (FACS) (Ekman & Friesen, 1978). FACS provides a detailed description of the muscular basis and outward manifestation of each expression in terms of so‐called action units (AUs). In the current study, crucial AUs were AU12 (pulling up lip corner; portrayal of happiness), AU4 (lowering eyebrows; anger, sadness, fear), AU1 (raising inner part of eyebrow; fear, sadness), and AU2 (raising outer part of eyebrow; fear) (cf. Deschamps et al., 2012; Ekman, 1979). The actors were trained under supervision of a certified FACS coder (author RZ). Besides the videos of dynamic emotional expressions, a 5‐s video was made of a neutral expression produced by each actor.

Videos of the dynamic facial expressions were evaluated by 15 male adolescents from the normal population (aged 11–16 years; mean age 11.5 years) and 14 female university students (aged 20–46 years; mean age 24.9 years). Emotional expressions were correctly identified in 90.8% of cases by males and 98.2% by females. Figures for correct identification of emotions were 96.7% for happiness, 95.0% for anger, 98.3% for sadness, and 88.1% for fear.

Still pictures of full facial expressions of the four emotions were also presented (Matsumoto & Ekman, 1988), each particular emotion being depicted by two female and two male models. Each picture was presented for 3 s and preceded by a 3‐s neutral expression by the same model with a 0.5‐s interval between pictures.

### 2.3 Experimental procedure

The experiment started with a 5‐min excerpt of an aquatic video (*Coral Sea Dreaming*, Small World Music Inc.) to induce a state of relaxation (Piferi et al., 2000). Next, participants were instructed to look at the videos of facial expressions. First, the four video's of neutral expressions were presented in a random order with intervals randomly varying between 1.5 and 2 s. After a pause of 8 s, eight videos (the four emotions each being displayed by a female and a male actor) were presented in a random order, each particular video being presented four times in succession. All 32 videos were presented with intervals randomly varying between 1.5 and 2 s. Thereupon, participants were instructed to look at still pictures of facial expressions. Sixteen pairs of pictures (four emotions depicted by four models), each pair consisting of a neutral and a full emotional expression by the same model, were presented in a random order with intervals between pairs randomly varying between 1.5 and 2 s. The instructions preceding videos and pictures implied just watching the stimuli attentively, avoiding any task demands like identification of gender or emotion.

### 2.4 Facial EMG recordings and quantification

EMG was bipolarly recorded from the left frontalis (involved in AU1 and AU2), corrugator supercilii (AU4), and zygomaticus major (AU12) muscles using surface Ag/AgCl electrodes (contact area 2 mm diameter, 15 mm distance between electrode centers). EMG signals were antialiasing filtered using a 512‐Hz lowpass filter, digitized at a rate of 1024 Hz, 20‐Hz digitally high‐pass filtered to remove low‐frequency artifacts (van Boxtel, 2001), and bandreject filtered (48–52 Hz) to remove 50‐Hz power line interference. EMG responses were visually inspected for remaining technical artifacts or strong potentials caused by disruptive actions like coughing, sneezing, strong eyeblinks, etcetera. For dynamic expressions, 2.6%, 2.6%, and 1.4% of EMG responses had to be discarded for corrugator, zygomaticus, and frontalis, respectively. For static expressions, these figures were 1.3%, 1.9%, and 1.3%.

EMG responses to videos of dynamic or static emotional expressions produced by the actors were quantified by calculating mean rectified EMG activity during subsequent 100‐ms periods. The duration of this period was based on a study showing that mechanical changes during fast frontalis contractions (as apparent from the so‐called mechanomyogram) rapidly decline at frequencies above 10 Hz (Alves & Chau, 2010). In case of dynamic expressions, raw EMG values were standardized by expressing them as a percentage of mean rectified EMG activity during the 2‐s neutral expression baseline period preceding the emotional expression. Standardized values were first averaged across the four presentations of the same emotion by the same actor and subsequently across the two actors expressing this emotion. If an EMG response to a dynamic expression had to be discarded due to artifacts, responses were averaged across the remaining two or three stimuli presented by the same actor. EMG responses during videos of neutral expressions were expressed as a percentage of mean rectified EMG during the entire 5‐s duration of the video.

Regarding still pictures of full facial expressions, raw EMG values during 100‐ms periods of emotional expressions were expressed as a percentage of mean rectified EMG activity during the preceding neutral expression. Subsequently, percentages were averaged across the four models expressing the same emotion. If a response had to be discarded, responses were averaged across the remaining three models.

### 2.5 Statistical analysis

Regarding dynamic expressions, mean EMG activity during the 500‐ms dynamic phase of expression was pairwise compared between the three subject groups using *t*‐tests for independent samples assuming equal or unequal variances. To apply these analyses with a greater temporal resolution, group comparisons were also performed for separate 100‐ms periods within the 500‐ms dynamic phase. Group comparisons were also performed for mean EMG activity level during the subsequent 1500‐ms apex of expression.

Based on studies demonstrating weak recognition of emotional facial expressions (Dawel et al., 2012; Muñoz, 2008), or weak amygdala responses to such expressions (Viding et al., 2012), in high CU groups, we expected smaller EMG responses in CU+ than in CU− or NC groups. We did not expect different EMG responses between CU− and NC because these two groups had comparable scores on the CU dimension (de Wied et al., 2012). Given these expectations, we applied a priori comparisons between pairs of groups given the advantages of such comparisons in terms of statistical power compared with performing analysis of variance with orthogonal follow‐up comparisons between groups (Ruxton & Beauchamp, 2008; Thompson, 1987). Homoscedastic or heteroscedastic one‐tailed *t*‐tests were thus performed for comparisons between CU+ and CU− or NC, and two‐tailed tests for comparisons between CU− and NC. EMG responses are reported insofar as a muscle is involved in a specific emotional expression, that is, zygomaticus in happy expressions, corrugator in angry, sad, or fearful expressions, and frontalis in sad or fearful expressions. Corrugator responses to happy expressions were also investigated because this muscle shows diminished activity relative to baseline during happy expressions (Deschamps et al., 2012; de Wied et al., 2006; Rymarczyk et al., 2011; Sato et al., 2008).

Similarly to dynamic expressions, EMG activity during the first 500‐ms of the 3‐s still pictures was compared between groups. Such comparisons were also made during the subsequent 2.5‐s period of the stimulus.

## 3 RESULTS

### 3.1 Dynamic facial expressions

**FIGURE 1:**
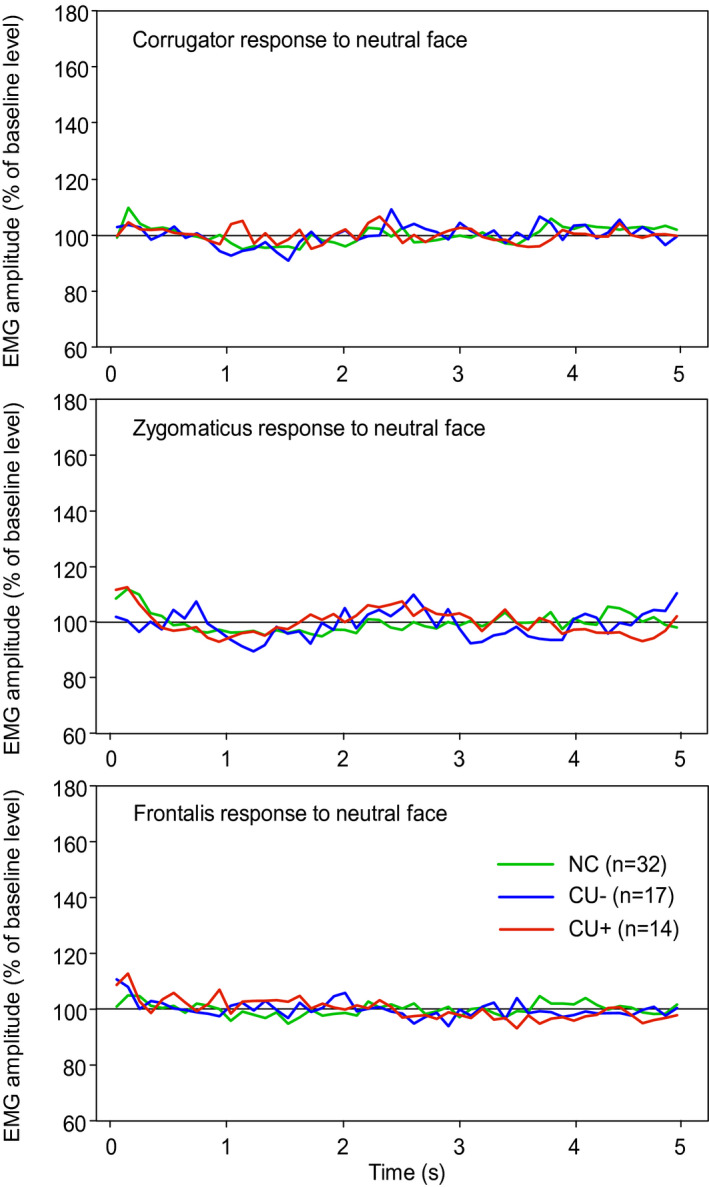
Mean EMG responses during 5‐s videos of neutral facial expressions. NC, normal control group; CU−, participants with low callous‐unemotional traits; CU+, participants with high callous‐unemotional traits

**FIGURE 2:**
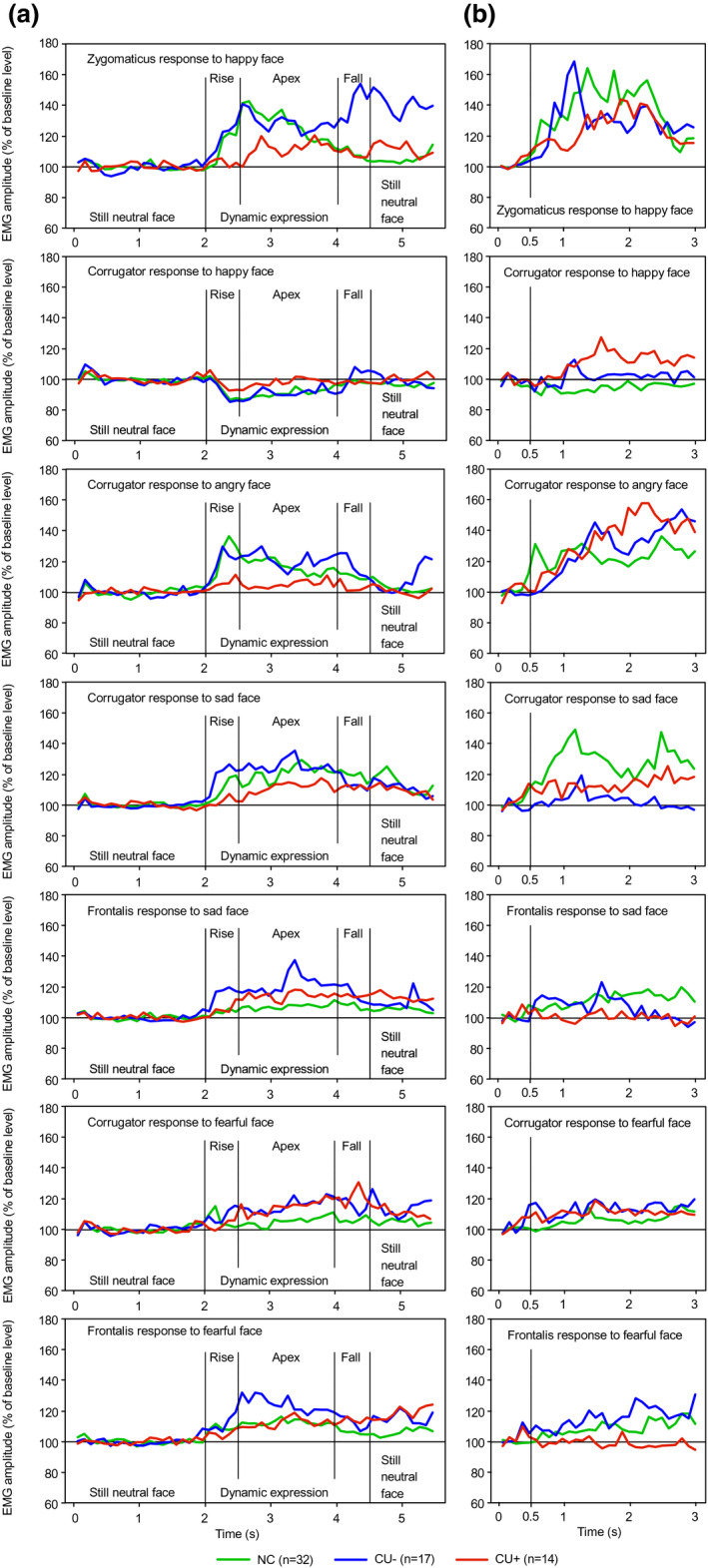
(a) Mean EMG responses during 5.5‐s videos of dynamic emotional facial expressions. (b) Mean EMG responses during 3‐s presentations of still pictures of emotional facial expressions. NC, normal control group; CU−, participants with low callous‐unemotional traits; CU+, participants with high callous‐unemotional traits

No group showed clear EMG responses to videos of neutral expressions (Figure 1). Several group differences in facial mimicry responses were observed during the 500‐ms rise time of dynamic expressions (Figure 2a). During happy expressions, the overall increase in zygomaticus activity was significantly weaker in the CU+ group than in CU− (*t*_19_ = −2.19, *p* = .021, Cohen's *d* = 1.00) or NC groups (*t*_44_ = −2.48, *p* = .008, *d* = 0.75) whereas CU− and NC did not differ (*t*_22_ = 0.88, *p* = .387, *d* = 0.38). On a more fine‐grained time scale, significant group differences in increased zygomaticus activity were already observed as early as 200–300 ms following stimulus onset (Table 1). During happy expressions, corrugator showed an overall inhibition of activity during the 500‐ms rise time which was significantly weaker for CU+ than for CU− (*t*_29_ = 2.27, *p* = .016, *d* = 0.84) or NC (*t*_40_ = 1.73, *p* = .046, *d* = 0.55) whereas CU− and NC did not differ (*t*_47_ = −0.73, *p* = .471, *d* = 0.21). On a more fine‐grained time scale, significant group differences were not observed earlier than 400–500 ms following stimulus onset (Table 1).

**TABLE 1:** Mean EMG responses during 100‐ms intervals of the dynamic phase of emotional facial expressions

Interval (ms)	0–100	100–200	200–300	300–400	400–500
*Zygomaticus response to happy face*					
CU+ (% baseline)	102.9	105.0	107.0	99.6	102.6
CU− (% baseline)	108.9	110.4	122.7	123.7	127.6
NC (% baseline)	99.9	101.8	118.7	121.9	120.5
CU+ vs. CU−	*t*_29_ = −1.33^+^ *d* = 0.29	*t*_27_ = −0.72 *d* = 0.28	*t*_27_ = −2.10* *d* = 0.81	*t*_18_ = −1.92* *d* = 0.91	*t*_41_ = −1.75* *d* = 0.55
CU+ vs. NC	*t*_19_ = 1.05 *d* = 0.48	*t*_44_ = 0.76 *d* = 0.48	*t*_38_ = −1.97* *d* = 0.64	*t*_44_ = −3.29*** *d* = 0.99	*t*_41_ = −2.74** *d* = 0.86
CU− vs. NC	*t*_20_ = 2.46* *d* = 1.10	*t*_20_ = 1.30 *d* = 0.58	*t*_18_ = 0.24 *d* = 0.11	*t*_24_ = 0.13 *d* = 0.05	*t*_21_ = 0.47 *d* = 0.21
*Corrugator response to happy face*					
CU+ (% baseline)	105.85	97.97	93.89	89.38	92.98
CU− (% baseline)	100.62	97.03	90.38	85.22	86.08
NC (% baseline)	102.00	97.82	92.07	86.76	87.70
CU+ vs. CU−	*t*_29_ = 1.71* *d* = 0.64	*t*_41_ = 0.37 *d* = 0.12	*t*_29_ = 1.22 *d* = 0.45	*t*_29_ = 1.42^+^ *d* = 0.53	*t*_41_ = 2.18* *d* = 0.68
CU+ vs. NC	*t*_44_ = 1.79* *d* = 0.54	*t*_44_ = 0.06 *d* = 0.02	*t*_44_ = 0.59 *d* = 0.18	*t*_44_ = 0.88 *d* = 0.27	*t*_44_ = 1.74* *d* = 0.52
CU− vs. NC	*t*_24_ = −0.58 *d* = 0.24	*t*_47_ = −0.31 *d* = 0.09	*t*_47_ = −0.63 *d* = 0.12	*t*_47_ = −0.55 *d* = 0.16	*t*_47_ = −0.53 *d* = 0.15
*Corrugator response to angry face*					
CU+ (% baseline)	101.69	104.40	105.44	105.66	111.20
CU− (% baseline)	105.85	115.65	129.55	123.29	121.19
NC (% baseline)	104.94	110.03	128.35	136.25	130.30
CU+ vs. CU−	*t*_29_ = −1.16 *d* = 0.43	*t*_21_ = −1.95* *d* = 0.85	*t*_24_ = −2.79** *d* = 1.14	*t*_23_ = −2.36* *d* = 0.98	*t*_26_ = −1.36^+^ *d* = 0.53
CU+ vs. NC	*t*_44_ = −1.39^+^ *d* = 0 42	*t*_41_ = −1.67^+^ *d* = 0.52	*t*_42_ = −3.52*** *d* = 1.09	*t*_39_ = −3.39*** *d* = 1.09	*t*_43_ = −2.38* *d* = 0.73
CU− vs. NC	*t*_23_ = 0.31 *d* = 0.13	*t*_24_ = 0.95 *d* = 0.39	*t*_47_ = 0.14 *d* = 0.04	*t*_46_ = −1.20 *d* = 0.35	*t*_45_ = −0.97 *d* = 0.29
*Corrugator response to sad face*					
CU+ (% baseline)	99.98	99.68	102.75	107.12	102.35
CU− (% baseline)	108.09	120.89	122.55	126.35	122.22
NC (% baseline)	101.86	104.24	106.57	117.74	119.20
CU+ vs. CU−	*t*_20_ = −1.29 *d* = 0.58	*t*_19_ = −2.96** *d* = 1.36	*t*_20_ = −2.10* *d* = 0.94	*t*_20_ = −1.75* *d* = 0.78	*t*_18_ = −2.38* *d* = 1.12
CU+ vs. NC	*t*_44_ = −0.80 *d* = 0.24	*t*_38_ = −1.59^+^ *d* = 0.52	*t*_44_ = −0.86 *d* = 0.26	*t*_44_ = −2.00* *d* = 0.60	*t*_44_ = −3.94*** *d* = 1.19
CU− vs. NC	*t*_17_ = 1.04 *d* = 0.50	*t*_19_ = 2.31* *d* = 1.06	*t*_19_ = 1.74* *d* = 0.80	*t*_19_ = 0.79 *d* = 0.36	*t*_23_ = 0.34 *d* = 0.14
*Frontalis response to sad face*					
CU+ (% baseline)	100.31	105.36	104.53	103.69	111.80
CU− (% baseline)	103.99	116.78	117.62	119.65	117.10
NC (% baseline)	100.58	103.59	102.73	105.87	103.62
CU+ vs. CU−	*t*_27_ = −1.14 *d* = 0.44	*t*_29_ = −2.54** *d* = 0.94	*t*_29_ = −2.67** *d* = 0.99	*t*_29_ = −2.80** *d* = 1.04	*t*_29_ = −1.00 *d* = 0.37
CU+ vs. NC	*t*_44_ = −0.10 *d* = 0.03	*t*_44_ = 0.49 *d* = 0.15	*t*_44_ = 0.49 *d* = 0.15	*t*_44_ = −0.52 *d* = 0.16	*t*_44_ = 1.89 *d* = 0.57
CU− vs. NC	*t*_47_ = 1.12 *d* = 0.33	*t*_47_ = 3.52*** *d* = 1.03	*t*_41_ = 3.92*** *d* = 1.22	*t*_47_ = 3.25** *d* = 0.95	*t*_18_ = 1.43 *d* = 0.67
*Corrugator response to fearful face*					
CU+ (% baseline)	100.78	99.01	101.44	105.48	105.69
CU− (% baseline)	108.33	104.25	104.53	112.37	115.24
NC (% baseline)	109.34	114.98	104.77	104.54	101.28
CU+ vs. CU−	*t*_21_ = −1.87* *d* = 0.82	*t*_29_ = −1.26 *d* = 0.47	*t*_29_ = −0.77 *d* = 0.29	*t*_23_ = −1.30 *d* = 0.54	*t*_27_ = −1.38^+^ *d* = 0.53
CU+ vs. NC	*t*_39_ = −3.81*** *d* = 1.22	*t*_42_ = −4.24*** *d* = 1.31	*t*_44_ = −0.72 *d* = 0.22	*t*_44_ = 0.22 *d* = 0.07	*t*_41_ = 0.90 *d* = 0.28
CU− vs. NC	*t*_22_ = −0.25 *d* = 0.11	*t*_47_ = −2.31* *d* = 0.67	*t*_47_ = −0.05 *d* = 0.01	*t*_47_ = 1.27 *d* = 0.37	*t*_24_ = 2.17* *d* = 0.89
*Frontalis response to fearful face*					
CU+ (% baseline)	105.70	105.07	101.58	104.61	108.43
CU− (% baseline)	107.45	109.50	106.39	114.69	119.00
NC (% baseline)	107.78	111.55	108.05	107.54	108.50
CU+ vs. CU−	*t*_22_ = −0.26 *d* = 0.11	*t*_17_ = −0.76 *d* = 0.37	*t*_29_ = −1.81* *d* = 0.67	*t*_29_ = −2.96** *d* = 1.10	*t*_29_ = −2.07* *d* = 0.77
CU+ vs. NC	*t*_44_ = −0.44 *d* = 0.13	*t*_36_ = −1.67 *d* = 0.56	*t*_44_ = −2.49** *d* = 0.75	*t*_44_ = −0.97 *d* = 0.29	*t*_44_ = −0.02 *d* = 0.01
CU− vs. NC	*t*_23_ = −0.05 *d* = 0.02	*t*_47_ = −0.31 *d* = 0.09	*t*_47_ = −0.64 *d* = 0.19	*t*_47_ = 2.28* *d* = 0.67	*t*_19_ = 1.25 *d* = 0.57

^+^ *p* < .10;

* *p* < .05;

** *p* < .01;

*** *p* <. 001.

During angry expressions, the overall increase in corrugator activity during the 500‐ms rise time was significantly weaker for CU+ than for CU− (*t*_19_ = −2.52, *p* = .010, *d* = 1.16) or NC (*t*_37_ = −3.45, *p* = .001, *d* = 1.13) whereas CU− and NC did not differ (*t*_47_ = −0.40, *p* = .693, *d* = 0.12). Following stimulus onset, these group differences specifically occurred as early as 100–200 ms for the difference between CU+ and CU−, and 200–300 ms for CU+ versus NC (Table 1).

During sad expressions, the overall increase in corrugator activity during the 500‐ms rise time was significantly weaker for CU+ than for CU− (*t*_18_ = −2.37, *p* = .015, *d* = 1.12) or NC (*t*_42_ = −1.87, *p* = .034, *d* = 0.58) whereas CU− and NC did not differ (*t*_47_ = 1.40, *p* = .168, *d* = 0.41). The difference between CU+ and CU− occurred as early as 100–200 ms following stimulus onset whereas the difference between CU+ and NC occurred not earlier than 300–400 ms following stimulus onset. In addition, activity in CU− was unexpectedly larger than in NC during the 100–300 ms period (Table 1).

During sad expressions, the overall increase in frontalis activity during the 500‐ms rise time did not differ between CU+ and NC (*t*_44_ = 0.59, *p* = .779, *d* = 0.18) and there was a tendency toward a significantly smaller overall increase in CU+ than in CU− (*t*_25_ = −1.55; *p* = .067, *d* = 0.62). On a time scale with a resolution of 100 ms, however, the increase in CU+ was significantly smaller than in CU− during the 100–400 ms period (Table 1). There was also an overall tendency toward a significantly larger response in CU− relative to NC (*t*_19_ = 2.07; *p* = .052, *d* = 0.95). This difference obtained significance during the 100–400 ms period (Table 1).

During fearful expressions, the overall increase in corrugator activity during the 500‐ms rise time did not differ between CU+ and NC (*t*_42_ = −0.89, *p* = .189, *d* = 0.27) although in the 0–100 ms and 100–200 ms intervals, activity was significantly larger in NC than in CU+ (Table 1). There was only a tendency toward a significantly weaker increase in CU+ than in CU− (*t*_29_ = −1.62, *p* = .058, *d* = 0.60). Responses in CU− and NC did not differ (*t*_47_ = 0.36, *p* = .723, *d* = 0.11).

During fearful expressions, the overall increase in frontalis activity during the 500‐ms rise time did not differ between CU+ and CU− (*t*_19_ = −1.24, *p* = .115, *d* = 0.57), although on a time scale with a resolution of 100 ms, activity was larger in CU− than in CU+ during the 200–500 ms period (Table 1). The overall increase in activity did not differ between CU+ and NC (*t*_42_ = −1.15, *p* = .128, *d* = 0.35), nor between CU− and NC (*t*_47_ = 0.52, *p* = .606, *d* = 0.15).

During the 1500‐ms apex of dynamic expressions, the large majority of EMG responses to the various expressions did not show significant group differences (*p* values ≥ .115) There was only one exception: during happy expressions, CU+ showed a significantly smaller inhibition of corrugator activity than NC (*t*_44_ = 1.78, *p* = .041, *d* = 0.54). Moreover, there were several tendencies toward significant group differences in the expected direction. During happy expressions, there was a tendency toward a significantly smaller increase in zygomaticus activity in CU+ compared with NC (*t*_41_ = −1.53, *p* = .067, *d* = 0.48), and a smaller inhibition of corrugator activity in CU+ than in CU− (*t*_29_ = 1.45, *p* = .080, *d* = 0.54). During angry expressions, CU+ showed a tendency toward a significantly smaller increase in corrugator activity than CU− (*t*_19_ = −1.66, *p* = .057, *d* = 0.76) or NC (*t*_38_ = −1.38, *p* = .088, *d* = 0.45) groups.

### 3.2 Static facial expressions

During the initial 500‐ms period of the 3‐s presentations of static expressions, CU+ did not show significantly different EMG responses to the various expressions compared with CU− or NC (Figure 2b). However, CU− and NC showed two response differences. During angry expressions, corrugator activity was significantly smaller in CU− than in NC (*t*_41_ = −2.04, *p* = .048, *d* = 0.64). During sad expressions, corrugator activity was smaller in CU− than in NC (*t*_47_ = −2.72, *p* = .009, *d* = 0.79).

During the subsequent 2.5‐s period of static expressions, frontalis activity during sad expressions was significantly smaller in CU+ than in NC (*t*_44_ = −1.91, *p* = .031, *d* = 0.58). During fearful expressions, frontalis activity was smaller in CU+ than in CU− (*t*_20_ = −2.44, *p* = .012, *d* = 1.09) or NC (*t*_40_ = −1.73, *p* = .046, *d* = 0.55).

## 4 DISCUSSION

As indicated in the introduction, the role of facial mimicry in emotional contagion and subsequent emotion recognition is not unequivocal, particularly not within the context of social interactions. Nevertheless, besides such social mimicry responses with relatively long response latencies, our study demonstrated the occurrence of short‐latency responses, which might play a role in early recognition of fast dynamic emotional expressions during daily life. Such responses may have a diagnostic value with regard to psychopathy because of the involvement of the amygdala, particularly in the case of dynamic emotional facial expressions. As outlined in the introduction, the amygdala is involved in early responses to emotional expressions. Therefore, we studied early mimicry responses to posed maximal dynamic expressions with a duration of 500 ms and a large degree of naturalness. We also studied mimicry responses to static emotional expressions, that is, the apex of dynamic expressions and still pictures of emotional expressions. Aside from performing group comparisons between responses across the entire 500‐ms dynamic expression period, we also performed such comparisons for separate 100‐ms periods during the various expressions to find out whether specific short‐latency responses differed between groups.

Adolescents with high CU traits generally showed smaller facial EMG responses to dynamic emotional facial expressions than participants with low CU traits or normal controls, particularly during happy, angry, and sad expressions. Such differences could often be observed already at short latencies (100 or 200 ms) following dynamic stimulus onset. Smaller responses in CU+ did generally not occur during the apex of dynamic expressions. Neither did they occur during the first 500 ms or the entire period of still picture presentation. We therefore conclude that specifically responses to dynamic expressions were subnormal in the CU+ group. Our results are in agreement with the conclusion that children and adolescents with CU traits show an impairment in recognizing emotional cues from other people (Blair et al., 2014). In addition, our results are consistent with the finding that reduced emotional responsiveness in DBD individuals with high relative to low CU traits is mostly demonstrated (a) with physiological measures, (b) in older (i.e., adolescent) samples, and (c) within an other‐oriented context (Northam & Dadds, 2020).

Although our conclusion holds for dynamic expressions of happiness, anger, and sadness, it does not apply to expressions of fear, which did not induce subnormal corrugator or frontalis responses in CU+. This seems paradoxical as the amygdala is sensitive to coarse visual input caused by wide‐open eyes in fearful faces (Vuilleumier et al., 2003; Whalen et al., 2004). However, the lack of differences between CU+ and NC or CU− could hypothetically be explained by a suppression of facial responses to fearful stimuli in the last two groups. The amygdala particularly responds to fearful facial expressions (Calder et al., 1996; Costafreda et al., 2008; Whalen et al., 2001). The central nucleus of the amygdala, being a nodal point of intra‐amygdalar circuits, is involved in fearful responses and projects to the ventrolateral periaqueductal grey area (VLPAG) in the midbrain (Bandler & Shipley, 1994). This area is involved in somatic and autonomic defensive responses to threatening stimuli (Cattaneo & Pavesi, 2014). VLPAG directly or indirectly projects to facial motoneurons (Holstege, 2002). Activity in VLPAG induced by a threatening stimulus mediates a passive coping strategy avoiding both tonic and phasic motor responses and leading to a complete immobility (Walker & Carrive, 2003). This absence of behavioral responses might explain the relatively small facial EMG responses to fearful faces in NC and CU− groups, thereby nullifying the expected difference with the CU+ group.

As explained in the introduction, early mimicry responses to dynamic emotional facial expressions may be considered automatic, preconscious responses that are difficult to suppress. If they would play a role in recognizing emotional facial expressions of other people, they necessarily should have a short latency to follow the natural dynamics of facial expressions occurring during normal human interactions. As revealed in the introduction, impaired recognition of emotional facial expressions by persons with psychopathic traits may be related to smaller amygdala responses, independent of the amount of attention devoted to the stimuli. We found that the CU+ group showed diminished early facial mimicry responses to dynamic happy, angry, or sad expressions whereas responses to static expressions were not abnormal. The latter agrees with a recent population‐based community study in adolescents demonstrating that amygdala responses to still pictures of happy, angry, sad, or fearful faces were not related to CU traits (Dotterer et al., 2020).

Subnormal mimicry responses to dynamic emotional facial expressions in the CU+ group might negatively influence recognition of such expressions during normal social interaction when dynamic expressions are prevalent and therefore crucial for emotion recognition. Subnormal mimicry may be related to smaller amygdala activation, consistent with the observation that amygdala activation is also smaller when facial muscle contractions are blocked by botulinum toxin (Hennenlotter et al., 2009).

It may be questioned whether subnormal mimicry responses to dynamic emotional expressions in the CU+ group—such mimicry responses being earlier designated as “motor empathy” (Blair, 2005)—are related to a lack of emotional empathy since emotional empathy requires that signals of emotional distress, like negative emotional facial expressions, are recognized (Blair, 2005; Blair et al., 2014). Both deficiencies might be based on a common amygdala dysfunction. In healthy persons, individual differences in the amplitude of short‐latency mimicry responses to dynamic or static emotional facial expressions appeared to be positively related to differences in emotional empathy (Dimberg et al., 2011; Dimberg & Thunberg, 2012; Rymarczyk et al., 2016).

A limitation of this study might be that we used videos of acted rather than spontaneous dynamic expressions. However, healthy persons who were able to distinguish acted from spontaneous dynamic smiles showed similar EMG mimicry responses to both types of stimuli (Krumhuber et al., 2014), suggesting that at amygdalar level there is no distinction between both types of expressions.

Another limitation might be that videos of specific emotional expressions were presented four times in succession rather than in a random order. In healthy persons, habituation of amygdala fMRI responses to successive presentations of pictures of emotional faces was repeatedly reported (Breiter et al., 1996; Fischer et al., 2003; Thomas et al., 2001; Whalen et al., 1998, 2001; Wright et al., 2001; Zald, 2003). Amygdala responses to film clips of emotional facial expressions did not show habituation if participants explicitly payed attention to the expression as part of a cognitive or motor task (van der Gaag et al., 2007). Nevertheless, we earlier observed habituation of corrugator EMG responses with repeated presentation of dynamic happy or angry faces in boys with DBD (de Wied et al., 2006).

Although videos and still pictures were presented in separate sections of the experiment and were each preceded by specific instructions, it remains uncertain whether EMG responses to static expressions could have been influenced by prior presentation of dynamic expressions.

## 5 CONCLUSION

In conclusion, male adolescents with DBD and high CU traits showed significantly smaller short‐latency facial mimicry responses to dynamic facial expressions of positive or negative emotions compared with boys with low CU traits or normal controls, whereas the last two groups did not differ in this respect. Mimicry responses to static facial expressions generally did not differ between groups. The subnormal dynamic mimicry responses in the high CU group may have negative consequences for human interactions during daily life since during such interactions dynamic rather than static expressions are common.

## AUTHOR CONTRIBUTIONS

**Anton van Boxtel:** Conceptualization; Data curation; Formal analysis; Investigation; Methodology; Project administration; Resources; Software; Supervision; Validation; Visualization; Writing‐original draft; Writing‐review & editing. **Ruud Zaalberg:** Methodology. **Minet de Wied:** Conceptualization; Data curation; Funding acquisition; Investigation; Methodology; Project administration; Resources; Supervision; Validation; Writing‐review & editing.
